# Response by Sharp to: “Ozempic Face” in Plastic Surgery: A Systematic Review of the Literature on GLP-1 Receptor Agonist Mediated Weight Loss and Analysis of Public Perceptions” by Daneshgaran et al

**DOI:** 10.1093/asjof/ojag080

**Published:** 2026-05-06

**Authors:** Gemma Sharp

I read with interest the article by Daneshgaran et al on glucagon-like peptide-1 (GLP-1) receptor agonists and the phenomenon popularly termed “Ozempic face” (Ozempic, Novo Nordisk, Bagsværd, Denmark).^1^ The authors provide a timely synthesis of early literature and report relevant perioperative considerations for aesthetic surgeons. However, the current framing also illustrates an emerging phenomenon in aesthetic medicine that may benefit from further conceptual clarification.

The framing of “Ozempic face” as a distinct clinical phenomenon may extend beyond the current evidence base. The studies included in the review are predominantly lower-level evidence, and there remains a lack of prospective or controlled data demonstrating a unique facial phenotype attributable specifically to GLP-1 receptor agonists.^1^ The changes described, facial volume loss and increased skin laxity, are well-recognized sequelae of rapid weight reduction and may not be specific to medication-assisted weight loss.^2^ In the absence of objective morphometric data or appropriate comparators, it may be helpful to interpret these findings with some caution when considering a distinct syndrome.

The use of Google Trends data (Google LLC, Mountain View, CA) to characterize public perception is a novel and valuable addition; however, it may warrant cautious interpretation. Although such data capture search behavior, they do not necessarily reflect clinical incidence. Increases in searches for terms such as “Ozempic face“ and “face filler” may therefore reflect the visibility and dissemination of terminology rather than the emergence of a new clinical condition. In this context, search trends may be more appropriately understood as indicators of cognitive salience rather than epidemiological signal.^3^

Finally, the adoption of the term “Ozempic face” within both media and clinical discourse raises a broader consideration regarding symptom attribution. Once established, such labels may influence how patients perceive and interpret expected post-weight-loss facial/bodily changes, with potential downstream effects on treatment-seeking behavior.^4,5^

Taken together, these considerations suggest value in approaching “Ozempic face” as an evolving construct, while encouraging future research incorporating longitudinal, objective facial assessment and patient-reported outcomes.
