# Correction: Response by Sharp to: “Ozempic Face” in Plastic Surgery: A Systematic Review of the Literature on GLP-1 Receptor Agonist Mediated Weight Loss and Analysis of Public Perceptions" by Daneshgaran et al

**DOI:** 10.1093/asjof/ojag174

**Published:** 2026-09-17

This is a correction to: Gemma Sharp, Response by Sharp to: “Ozempic Face” in Plastic Surgery: A Systematic Review of the Literature on GLP-1 Receptor Agonist Mediated Weight Loss and Analysis of Public Perceptions" by Daneshgaran et al, *Aesthetic Surgery Journal Open Forum*, Volume 8, 2026, ojag080, https://doi.org/10.1093/asjof/ojag080

The title of the originally published paper was errored in reading: "Response by Sharp to ““Ozempic Face”: Construct, Consequence, or Clinical Entity?” by Daneshgaran et al" and has been corrected in the paper now to read: "Response by Sharp to: "“Ozempic Face” in Plastic Surgery: A Systematic Review of the Literature on GLP-1 Receptor Agonist Mediated Weight Loss and Analysis of Public Perceptions" by Daneshgaran et al".

